# Cardioprotective Effects of Osteopontin-1 during Development of Murine Ischemic Cardiomyopathy

**DOI:** 10.1155/2014/124063

**Published:** 2014-05-29

**Authors:** Georg D. Duerr, Bettina Mesenholl, Jan C. Heinemann, Martin Zoerlein, Peter Huebener, Prisca Schneider, Andreas Feisst, Alexander Ghanem, Klaus Tiemann, Daniela Dewald, Armin Welz, Oliver Dewald

**Affiliations:** ^1^Department of Cardiac Surgery, University Clinical Centre Bonn, Sigmund-Freud-Straße 25, 53105 Bonn, Germany; ^2^Department of Pediatric Cardiology, University Clinical Centre Bonn, Sigmund-Freud-Straße 25, 53105 Bonn, Germany; ^3^Department of Medicine I, University Clinical Center Hamburg-Eppendorf, Martinistr. 52, 20251 Hamburg, Germany; ^4^Department of Radiology, University Clinical Centre Bonn, Sigmund-Freud-Straße 25, 53105 Bonn, Germany; ^5^Department of Medicine II and Cardiology, University Clinical Centre Bonn, Sigmund-Freud-Straße 25, 53105 Bonn, Germany; ^6^Isar Heart Center, Isar Kliniken, Sonnenstraße 24-26, 80331 Munich, Germany; ^7^Department of Anesthesiology and Intensive Care, University Clinical Centre Bonn, Sigmund-Freud-Straße 25, 53105 Bonn, Germany

## Abstract

Repetitive brief ischemia and reperfusion (I/R) is associated with ventricular dysfunction in pathogenesis of murine ischemic cardiomyopathy and human hibernating myocardium. We investigated the role of matricellular protein osteopontin-1 (OPN) in murine model of repetitive I/R. One 15-min LAD-occlusion followed by reperfusion was performed daily over 3, 5, and 7 consecutive days in C57/Bl6 wildtype- (WT-) and OPN^−/−^-mice (*n* = 8/group). After echocardiography hearts were processed for histological and mRNA-studies. Cardiac fibroblasts were isolated, cultured, and stimulated with TGF-**β**1. WT-mice showed an early, strong, and cardiomyocyte-specific osteopontin-expression leading to interstitial macrophage infiltration and consecutive fibrosis after 7 days I/R in absence of myocardial infarction. In contrast, OPN^−/−^-mice showed small, nontransmural infarctions after 3 days I/R associated with significantly worse ventricular dysfunction. OPN^−/−^-mice had different expression of myocardial contractile elements and antioxidative mediators and a lower expression of chemokines during I/R. OPN^−/−^-mice showed predominant collagen deposition in macrophage-rich small infarctions. We found lower induction of tenascin-C, MMP-9, MMP-12, and TIMP-1, whereas MMP-13-expression was higher in OPN^−/−^-mice. Cultured OPN^−/−^-myofibroblasts confirmed these findings. In conclusion, osteopontin seems to modulate expression of contractile elements, antioxidative mediators, and inflammatory response and subsequently remodel in order to protect cardiomyocytes in murine ischemic cardiomyopathy.

## 1. Introduction

Clinical and experimental studies provided evidence to establish the concept of repetitive episodes of brief ischemia and reperfusion (I/R) as an important mechanism in the development of ischemic heart disease [1, 2]. Repetitive I/R leads to progressive morphological and functional changes in human heart resulting in hibernating myocardium. Hibernating myocardium is a condition, where myocardial function is reduced under poor substrate availability in order to prevent cardiomyocyte loss. The hallmark of this condition is the reversibility of ventricular dysfunction upon restoration of coronary blood flow—reperfusion. The presence of newly recruited inflammatory cells in human hibernating myocardium has been shown to be beneficial for the restoration of left ventricular function after revascularization [3]. In order to better understand the underlying mechanisms, we developed a murine model of repetitive, brief I/R [4]. This model shares pathological and functional features of the human hibernating myocardium including a transient inflammatory reaction, interstitial fibrosis, and reversible left ventricular dysfunction. We described an important role for the chemokine CCL2 in development of interstitial fibrosis and impaired ventricular function in this model [5] and also found a strong induction of osteopontin during I/R.

Osteopontin is a matricellular protein and cytokine involved in the regulation of macrophage function and as a remodeling-associated mediator in different tissues [6]. Expression of osteopontin was associated with important fibrogenic signals during development of* bleomycin*-induced lung fibrosis [7]. Osteopontin expression was required for differentiation of myofibroblasts* in vitro* [8]. Osteopontin has also been associated with development of fibrosis and cardiac adaptation in angiotensin II-induced murine myocardial hypertrophy [9]. Mice lacking osteopontin revealed exaggerated left ventricular dilation and reduced collagen deposition after nonreperfused myocardial infarction [10]. On the other hand, cardiac-specific overexpression of osteopontin led to the development of dilated cardiomyopathy being lethal in mice at 12 weeks of age [11]. In a recent study, osteopontin has further been suggested to have a major influence on myocardial remodeling in desmin-deficient mice, which suffer from myocardial degeneration without any kind of injury [12]. In contrast to the published data, we investigated the role of osteopontin in development of ischemic cardiomyopathy using our model of repetitive, brief I/R without myocardial infarction.

We describe novel aspects of osteopontin-dependent cardioprotection in murine ischemic myocardium influencing expression of contractile elements and chemokines, as well as regulation of remodeling factors tenascin-C and matrix metalloproteinases (MMP).

## 2. Material and Methods

### 2.1. Study Animals

All mouse experiments were performed on 20–25 g and 10–16-week-old mice. We used wild type- (WT-) C57BL/6J mice (Charles River, Sulzfeld, Germany) and the homozygote osteopontin-1-deficient- (OPN^−^/^−^-) mice, which were derived on C57BL/6J-background and provided by Rittling [13]. All experiments were performed in accordance with an animal protocol approved by the local governmental authorities and according to the EU Directive 2010/63/EU for animal experiments.

### 2.2. Mouse Model of Repetitive I/R

Briefly, in an initial surgery, an 8-0 Prolene suture (Ethicon, Norderstedt, Germany) was placed around the left descending coronary artery and stored subcutaneously as previously described [4]. After a recovery period of 7 days following initial surgery trauma, repetitive I/R was performed. One daily episode of 15 min coronary artery occlusion was followed by reperfusion until the next day. This protocol was repeated over 1, 3, 5, or 7 consecutive days. Left ventricular function was assessed via echocardiography after 7 days I/R before heart retrieval for histology group. Hearts were excised and fixated in zinc-paraformaldehyde (Z-fix, 4%; Anatech, Battle Creek, MI, USA) for histology, stored in RNAlater (Qiagen, Hilden, Germany) for mRNA-studies, or dissolved for cardiomyocyte isolation.

### 2.3. Echocardiography

Mice were anesthetized with isoflurane under continuous heart rate monitoring to minimize cardiodepressive effects as previously described [4]. We used a commercially available ultrasound system (HDI-5000; ATL-Phillips, Oceanside, CA, USA) equipped with a linear array transducer (CL15-7) operating at 15 MHz and providing frame rates up to 284 Hz. Briefly, 2D-guided M-mode data was acquired in the parasternal short-axis view at the level of the papillary muscle. Fractional shortening as a global left ventricular function parameter and anterior wall thickening as a regional parameter of left ventricular function were calculated as previously described [4].

### 2.4. Histology and Immunohistochemistry

Basic histological evaluation was performed via haematoxylin and eosin staining. Picrosirius red staining was used to visualize collagen deposition and quantitative planimetric analysis of collagen stained area was performed as already published [4]. Immunohistochemical staining was performed using Vectastain Elite ABC kits and diaminobenzidine (AXXORA, Lörrach, Germany). We used the following antibodies: *α*-smooth muscle actin (*α*-smac; Sigma, Steinheim, Germany), monocyte-specific rat anti-mouse antibody F4/80 (Abcam, Cambridge, UK), tenascin-C rabbit anti-chicken polyclonal antibody (Chemicon, Temecula, CA, USA)* in vivo,* and troponin T (Fisher Scientific, Schwerte, Germany) and CD31 (BD-Pharmingen, Heidelberg, Germany)* in vitro*. F4/80-positive cells were counted manually in order to ascertain their monocyte/macrophage morphology.

### 2.5. Cardiomyocyte Isolation

We extracted cardiomyocytes from hearts after 3 days I/R using Langendorff-apparatus (Radnoti Technologies Inc., Monrovia, CA, USA) in order to measure the cardiomyocyte specific mRNA-expression of osteopontin as previously described [14]. Briefly, ischemic hearts were perfused with collagenase B (Roche, Mannheim, Germany) for digestion. The eluate containing the cardiomyocytes was subjected to manual cell count immediately after isolation. Cell count of troponin T stained cells revealed >90% of cells being cardiomyocytes. The isolated cells were transferred to Trizol (Invitrogen, Karlsruhe, Germany) for subsequent mRNA-isolation. The whole isolation protocol was performed within 20 min.

### 2.6. Cardiac Fibroblast Cell Culture

Fibroblasts were isolated from nonischemic mouse hearts according to a slightly modified protocol [15]. Briefly, three noninfarcted, naïve WT- or OPN^−^/^−^-hearts were pooled for one sample. Each group consisted of *n* = 5–7 pooled samples for further experiments. The hearts were dissected free of vessels and atria, transferred to 1 mL of collagenase buffer (Roche), and quickly minced into small pieces with scalpel and microforceps. This digestion process continued until no visible tissue fragments were left. Next, the cell suspensions of 3 mice were pelleted, washed, combined, and plated on a T75 tissue-culture flask (Invitrogen) in full medium supplemented with 10% of FBS (Invitrogen) and antibiotic/antimycotic solution (Invitrogen). After 24 h incubation, nonadherent cells were removed by discarding the supernatant. Upon reaching confluence, cells were detached with trypsin/EDTA (Invitrogen), split in a 1 : 2 or 1 : 4 ratio, and recultured. Characteristic fibroblast morphology was determined visually under a light microscope. Only fibroblasts at passages 2–4 were used for experiments. Pure fibroblast cultures were confirmed by immunocytochemistry using antibodies against *α*-smac, troponin T, F4/80, and CD31. Cells were stimulated with human TGF-*β*1 (100 ng/mL; PeproTech, Hamburg, Germany) and cultured under normoxia (21% O_2_, 5% CO_2_, 37°C) or hypoxic conditions (2% O_2_, 5% CO_2_, 37°C) for 6 and 24 h. At the end of the experiment, cells were harvested and stored in Trizol (Invitrogen) at −80°C until RNA extraction.

### 2.7. RNA-Extraction and Taqman RT-qPCR

The mRNA-expression was determined using Taqman real time quantitative PCR system (RT-qPCR, Applied Biosystems, Foster City, CA, USA). Total RNA was isolated with a standard protocol using phenole/chloroform extraction (Trizol, Invitrogen). First-strand cDNA was synthesized using the high capacity cDNA transcription kit (Applied Biosystems) with random hexameric primers as described in the manufacturer's protocol. cDNA was diluted 1/10 and analyzed in RT-qPCR according to the manufacturer's instructions on an ABI Prism 7900 HT Sequence Detection System using SDS2.2 Software (Applied Biosystems). Target gene-expression was normalized to an internal control and to GAPDH for* in vivo* experiments or 18 S for* in vitro* studies. All primers were measured using FAM-TAMRA chemistry and the relative standard curve method. Dissociation curve analysis was performed to ascertain the amplification of a single PCR product.

### 2.8. Statistical Analysis

Data were normally distributed and presented as mean ± S.E.M. Two way ANOVA with a Student's Newman-Keuls' corrected post hoc analysis was done to compare differences between the groups (Prism 5.0; Graph Pad, La Jolla, CA, USA). Differences with *P* ≤ 0.05 were considered significant.

## 3. Results

### 3.1. OPN^−^/^−^-Mice Develop Small, Nontransmural Infarctions during Repetitive I/R Episodes

**Figure 1:**
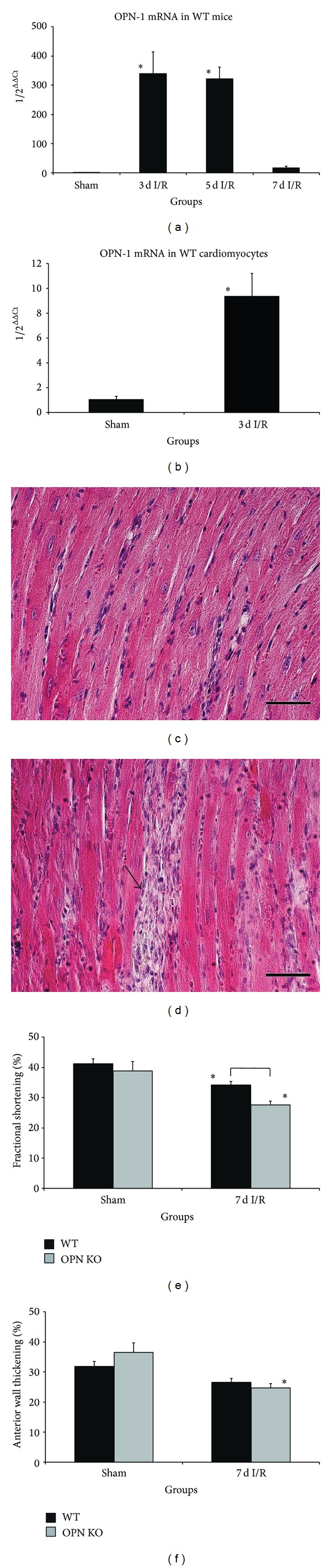
Osteopontin-deficiency leads to cardiomyocyte loss and poor left ventricular function. (a) Osteopontin mRNA-expression in whole WT-mouse hearts during repetitive I/R and (b) in Langendorff-isolated adult WT-cardiomyocytes after 3 days I/R compared to sham. (c) Representative HE-stained section of WT-heart after 7 days I/R shows interstitial cellular infiltration in contrast to (d) irreversible loss of cardiomyocytes in OPN^−^/^−^-mice (arrow). (e) Fractional shortening was impaired in both strains, but loss of left ventricular contractility was significantly reduced in OPN^−^/^−^-mice after 7 d I/R compared to WT. (f) Anterior wall thickening was significantly reduced in OPN^−^/^−^-mice after 7 days I/R. *n* = 8–11/group. Scale bars represent 50 *μ*m. RT-qPCR using Taqman and mRNA-expression is related to controls and GAPDH using comparative ΔΔCt-method. Bracket indicates *P* ≤ 0.05 between genotypes; ∗ indicates *P* ≤ 0.05 versus respective shams.

Brief repetitive I/R led to ~350-fold induction of osteopontin in WT-mice after 3 and 5 days of the ischemic protocol (Figure 1(a)). In order to investigate whether osteopontin was produced by cardiomyocytes in ischemic heart, we isolated cardiomyocytes after 3 days I/R from WT-hearts via Langendorff-perfusion and found a significant 9-fold increase in osteopontin mRNA-expression (Figure 1(b)). Naïve and sham operated OPN^−^/^−^-mice presented with normal myocardial morphology and cardiac function when compared with WT-mice, as previously described [10]. Repetitive I/R caused no loss of cardiomyocytes after 7 days I/R in WT-mice (Figure 1(c)), as previously reported [4]. In contrast, after 3 days I/R OPN^−^/^−^-mice showed an irreversible loss of cardiomyocytes in small areas of nontransmural infarctions and a mostly consolidated scar formation after 7 days I/R in these regions (Figure 1(d)). These small, nontransmural infarctions were scattered throughout the ischemic area of the left ventricular anterior wall. Echocardiography measurement of fractional shortening revealed significant dysfunction in both strains when compared to their shams (Figure 1(e)). The left ventricular dysfunction was also significantly worse in OPN^−^/^−^-mice compared to the WT-mice. Anterior wall thickening measurement showed comparable degree of regional dysfunction in ischemic anterior wall between both strains after 7 days I/R, but only ischemic OPN^−^/^−^-hearts demonstrated with significantly worse left ventricular function compared to their shams (Figure 1(f)).

### 3.2. Ischemic OPN^−^/^−^-Hearts Show Different Expression of MHC- and Actin-Isoforms and Antioxidative Mediators

**Figure 2:**
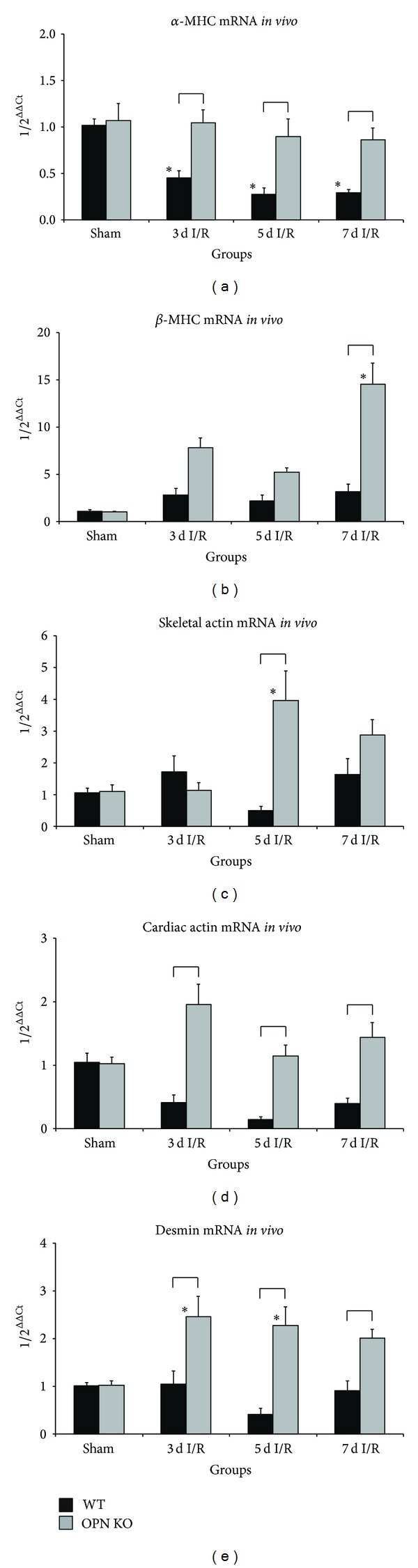
Ischemic OPN^−^/^−^-hearts show different expression contractile elements. mRNA-expression of (a) *α*-myosin heavy chain (*α*-MHC), (b) *β*-MHC, (c) skeletal actin, (d) cardiac actin, and (e) desmin in OPN^−^/^−^-mice compared to WT-mice during repetitive I/R. *n* = 8-9/group. RT-qPCR using Taqman and mRNA-expression is related to controls and GAPDH using comparative ΔΔCt-method. Bracket indicates *P* ≤ 0.05 between genotypes; ∗ indicates *P* ≤ 0.05 versus respective shams.

In order to investigate the underlying mechanisms for the irreversible loss of cardiomyocytes in OPN^−^/^−^-hearts, we measured myosin heavy chain (MHC) isoforms. OPN^−^/^−^-mice were unable to decrease expression of more ATP consuming and therefore energetically unfavorable *α*-MHC in contrast to WT-mice during repetitive I/R (Figure 2(a)). At the same time, OPN^−^/^−^-mice showed up to 5-fold higher expression of less ATP demanding *β*-MHC after 7 days I/R than WT-mice (Figure 2(b)). Despite of this adaptation, continuous expression of *α*-MHC might result in unfavorable utilization of ATP in ischemic osteopontin-deficient myocardium. We further investigated the contractile elements and measured mRNA-expression of skeletal and cardiac actin isoforms. While WT-hearts showed practically unchanged expression of skeletal actin during repetitive I/R, while their OPN^−^/^−^-mice counterparts experienced a significant ~4-fold upregulation after 5 days I/R, when compared to respective shams or WT-group (Figure 2(c)). The expression of cardiac specific actin was downregulated in WT-hearts to as low as 14% of sham expression level during repetitive I/R protocol (Figure 2(d)). Interestingly, OPN^−^/^−^-hearts showed a continuous and significant upregulation of cardiac actin when compared to the respective WT-groups. As an additional marker of cardiac remodeling, we measured mRNA expression of desmin and found no induction of it in WT-hearts, in contrast to a significantly higher expression of it (max. 2.5-fold) in OPN^−^/^−^-hearts (Figure 2(e)).

**Figure 3:**
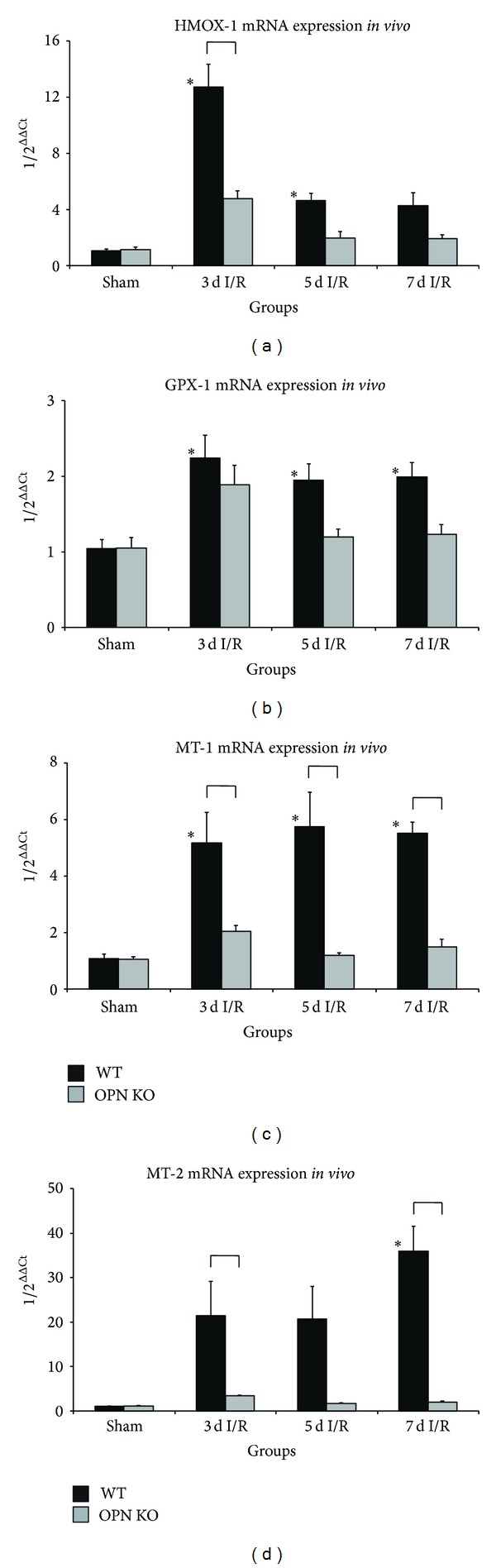
Ischemic OPN^−^/^−^-hearts show different expression of antioxidative mediators. mRNA-expression of antioxidative mediators (a) heme oxygenase- (HMOX-) 1 and (b) glutathione peroxidase- (GPX-) 1. mRNA-expression of zinc-storage proteins (c) metallothionein (MT)-1 and (d) MT-2. *n* = 8-9/group. RT-qPCR using Taqman and mRNA-expression is related to controls and GAPDH using comparative ΔΔCt-method. Bracket indicates *P* ≤ 0.05 between genotypes; ∗ indicates *P* ≤ 0.05 versus respective shams.

OPN^−^/^−^-mice showed a significant 3-fold lower mRNA-induction of antioxidative mediator heme oxygenase-1 after 3 days of I/R and a tendency towards less induction thereafter (Figure 3(a)). OPN^−^/^−^-mice also presented with a tendency to less induction of glutathione peroxidase-1 during I/R (Figure 3(b)). Furthermore, their expression of zinc-storage and antioxidative proteins metallothionein-1 and -2 was significantly decreased during the I/R-protocol (Figures 3(c) and 3(d)). These differences in expression of contractile elements and induction of antioxidative mediators seem to be the major factors responsible for the irreversible damage of the cardiomyocytes in remote perfusion areas, which histologically presented as small, nontransmural infarcted areas.

### 3.3. Differences in Cellular and Molecular Response during Postischemic Inflammation

**Figure 4:**
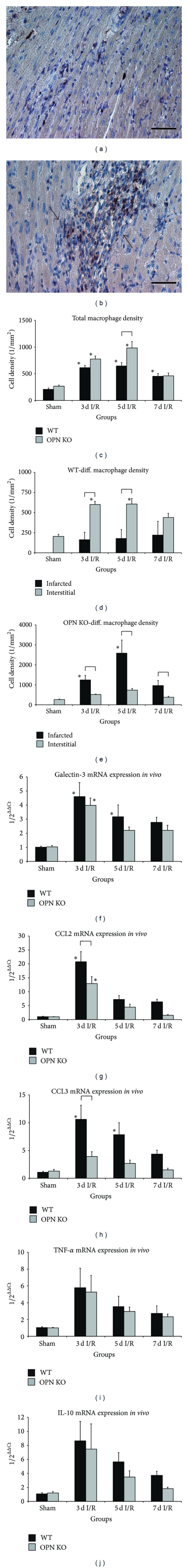
Cellular and molecular changes in response to I/R. (a) Representative section of left ventricle after 7 days I/R in a WT-heart shows low interstitial macrophage accumulation (F4/80). (b) High accumulation of macrophages in areas of small nontransmural infarctions in OPN^−^/^−^-hearts (arrows). (c) Cell density of F4/80 positive macrophages in WT- compared to OPN^−^/^−^-hearts during I/R. (d) Differential macrophage cell count in WT-hearts revealed only few cells within areas of small infarctions (infarcted) in comparison to interstitial space. (e) In contrast, significantly more cells invaded areas of small infarctions in OPN^−^/^−^-mice than interstitial space. mRNA-induction of the galectin-3 (f), chemokines (g) CCL2 and (h) CCL3, and cytokines (i) TNF-*α* and (j) IL-10 in OPN^−^/^−^-hearts compared to WT-hearts during repetitive I/R. *n* = 8–11/group. Scale bars represent 50 *μ*m. RT-qPCR using Taqman and mRNA-expression is related to controls and GAPDH using comparative ΔΔCt-method. Bracket indicates *P* ≤ 0.05 between genotypes; ∗ indicates *P* ≤ 0.05 versus respective shams.

In the next step we investigated inflammatory reaction and found a different pattern of macrophage distribution between the genotypes. Macrophages were evenly distributed in the interstitial space of ischemic WT-hearts after 7 days I/R (Figure 4(a)). In contrast, the OPN^−^/^−^-mice presented with increased macrophage infiltration into small, nontransmural infarctions (Figure 4(b)). The quantitative analysis of F4/80 staining revealed a comparable macrophage density after 3 and 7 days I/R in both genotypes (Figure 4(c)). Still, at the maximum of macrophage action after 5 days I/R we found a significant 46% higher cell density in OPN^−^/^−^-hearts than in WT-hearts. Further analysis of cellular localization showed a significantly stronger (2-3-fold) macrophage infiltration of the interstitial space in WT-hearts than in the very rarely observed small infarcted areas (Figure 4(d)). At the same time, OPN^−^/^−^-hearts demonstrated with a significant up to 3.5-fold higher macrophage density in small, nontransmural infarctions when compared to their interstitial space (Figure 4(e)). The mRNA-expression of galectin-3, also known as MAC-2, was significantly induced after 3 and 5 days I/R in WT-hearts, as well as after 3 days in OPN^−^/^−^-hearts, but not different between the two strains (Figure 4(f)). Interestingly, measurement of mRNA-expression of chemokines involved in this process revealed a significant 40% lower induction of macrophage-related CCL2 after 3 days I/R in OPN^−^/^−^-hearts (Figure 4(g)). Another macrophage-related chemokine, CCL4, showed the same induction pattern in both genotypes as CCL2 (data not shown). Neutrophils-related chemokine CCL3 presented also with a significant 60% lower induction in OPN-hearts than in WT-hearts (Figure 4(h)), thus suggesting that chemokines are not the critical mediators driving the inflammatory response in osteopontin-deficient ischemic hearts. Indeed, the mRNA-expression of proinflammatory cytokine TNF-*α* showed a comparable induction between the genotypes (Figure 4(i)), thus suggesting that TNF-*α* acts as a main chemotactic agent in ischemic OPN^−^/^−^-hearts. The resolution of inflammatory response was evaluated using mRNA-expression of anti-inflammatory cytokine IL-10 (Figure 4(j)). IL-10 expression profile was comparable between both genotypes, and this provides suitable environment for the subsequent onset of myocardial remodeling.

### 3.4. Attenuated Remodeling in Ischemic OPN^−^/^−^-Hearts

**Figure 5:**
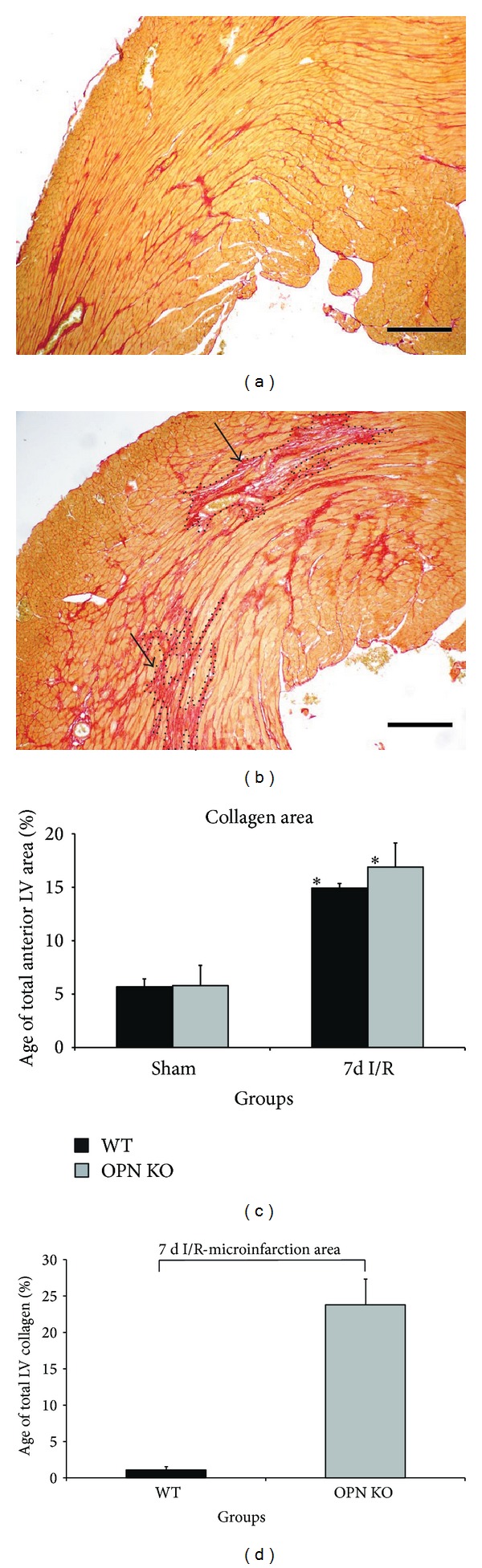
Collagen deposition in areas of small, nontransmural infarctions in OPN^−^/^−^-hearts during repetitive I/R. (a) Representative section of left ventricle in a WT-heart after 7 days I/R shows interstitial fibrosis in picrosirius red staining. (b) In contrast, OPN^−^/^−^-mice reveal relatively loose collagen deposition in areas of cardiomyocyte loss-small, nontransmural infarctions (arrows and dotted lines show confluent areas of patchy replacement fibrosis), but less interstitial collagen in the ischemic area. (c) Planimetrical analysis of collagen stained area between the genotypes after 7 days I/R. (d) Differential analysis of collagen deposition in small, nontransmural infarctions. *n* = 8–11/group. Scale bars represent 200 *μ*m. Bracket indicates *P* ≤ 0.05 between genotypes; ∗ indicates *P* ≤ 0.05 versus respective shams.

We investigated several aspects of myocardial remodeling* in vivo* and analyzed the function of osteopontin in fibroblasts* in vitro*. Picrosirius red staining of collagen in WT-mice showed an evenly distributed interstitial fibrosis, with thin, dense collagen strains after 7 days I/R throughout the ischemic area of the left ventricle (Figure 5(a)). OPN^−^/^−^-hearts presented at the same time point with marked collagen deposition in small areas of nontransmural infarctions, which were partially confluent and where the collagen appeared not yet to be compacted (upper black arrow, Figure 5(b)). This morphology is indicative for prolonged remodeling process. In order to quantify the extent of these confluent, patchy, small areas of nontransmural infarctions, we performed planimetric analysis of picrosirius red area and found comparable total collagen stained area as a percentage of the total left ventricular area between both genotypes after 7 days I/R, which was significantly higher (up to 3-fold) than in the respective shams (Figure 5(c)). The differential analysis of collagen deposition in these small infarctions revealed a significantly larger area of them in OPN^−^/^−^-hearts covering almost 25% of the total left ventricular area, when compared to only 2% observed in WT-hearts (Figure 5(d)).

**Figure 6:**
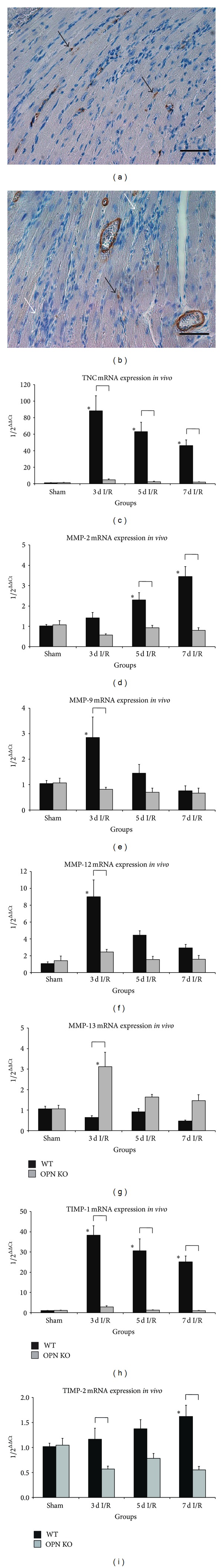
Attenuated remodeling in ischemic OPN^−^/^−^-hearts. (a) Representative section of left ventricle after 7 days I/R in a WT-heart shows numerous *α*-smac positive myofibroblasts (black arrows) in the ischemic myocardium. (b) At the same time point, *α*-smac positive signals (black arrow) were almost absent in small, nontransmural infarctions (white arrows) of OPN^−^/^−^-hearts. mRNA-induction of (c) tenascin-C (TNC), (d) MMP-2, (e) MMP-9, (f) MMP-12, (g) MMP-13, (h) TIMP-1, and (i) TIMP-2 in WT- and OPN^−^/^−^-hearts during repetitive I/R. *n* = 8–11/group. Scale bars represent 50 *μ*m. RT-qPCR using Taqman and mRNA-expression is related to controls and GAPDH using comparative ΔΔCt-method. Bracket indicates *P* ≤ 0.05 between genotypes; ∗ indicates *P* ≤ 0.05 versus respective shams.

Further analysis of cells and remodeling markers included visualization of myofibroblasts using *α*-smac staining. In contrast to numerous myofibroblasts found in ischemic area of the WT-myocardium (Figure 6(a)), we observed only very few myofibroblasts in ischemic OPN^−^/^−^-hearts (Figure 6(b)). The mRNA-expression of early remodeling marker tenascin-C was strongly induced in WT-hearts being ~18-fold higher than in OPN^−^/^−^-hearts (Figure 6(c)). Still, the OPN^−^/^−^-hearts presented with ~4-fold induction above the sham-level. The mRNA-expression of growth and myofibroblast differentiation factor TGF-*β*1 was comparable between the genotypes and slightly induced in both strains after 3 days I/R (data not shown). Another prominent and significant difference was found in the absence of induction of MMP-2 and MMP-9 in OPN^−^/^−^-hearts when compared to their induction in WT-samples (Figures 6(d) and 6(e)). Interestingly, MMP-12 expression was also significantly ~4-fold higher in WT-hearts after 3 days I/R when compared to the OPN^−^/^−^-hearts, but the OPN^−^/^−^-hearts revealed a nonsignificant ~2-fold induction when compared to their shams (Figure 6(f)). Apparently, the only enzyme involved in collagen deposition was MMP-13, which was significantly up to 3-fold higher induced in OPN^−^/^−^-hearts after 3 days I/R and followed by a tendency to a higher expression thereafter, when compared to the WT-hearts (Figure 6(g)). Interestingly, the expression of tissue-inhibitor of MMP (TIMP-1) (Figure 6(h)) and TIMP-2 (Figure 6(i)) was also significantly lower in OPN^−^/^−^-hearts during repetitive I/R than in WT-hearts. TIMP-4 presented a similar induction pattern as TIMP-2 for both genotypes (data not shown).

**Figure 7:**
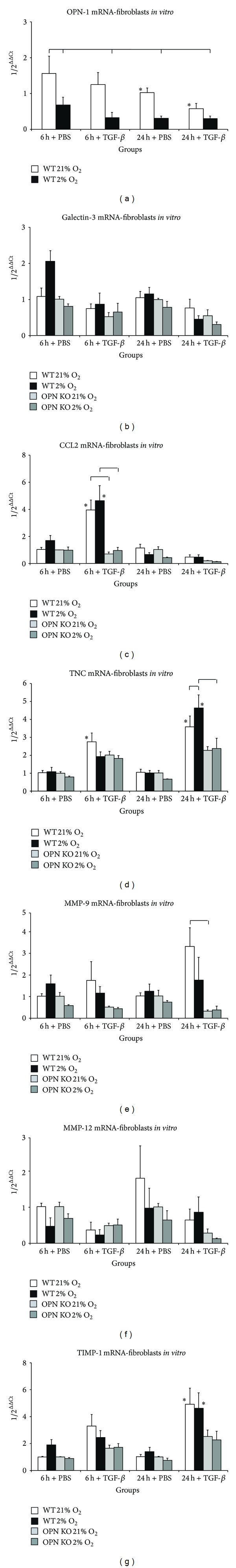
Expression of remodeling markers in myofibroblasts* in vitro*. Cultured myofibroblasts from WT- and OPN^−^/^−^-hearts were stimulated with human TGF-*β*1 or PBS as control under normoxia (21% O_2_) or hypoxia (2% O_2_). mRNA-expression of (a) OPN-1 in WT-cells only. mRNA-expression of (b) galectin-3, (c) CCL2, (d) tenascin-C (TNC), (e) MMP-9, (f) MMP-12, and (g) TIMP-1. *n* = 5–7/group. RT-qPCR using Taqman and mRNA-expression is related to controls and 18 S (*in vitro*) using comparative ΔΔCt-method. Bracket indicates *P* ≤ 0.05 between genotypes; ∗ indicates *P* ≤ 0.05 versus 6 h + PBS normoxia group (control).

In the last set of experiments we analyzed fibroblasts* in vitro*, which were derived from naïve hearts, cultured under normoxic or hypoxic conditions, and additionally stimulated with human TGF-*β*1 to differentiate into myofibroblasts. As a proof of principle, we measured the OPN-1 in WT-cells and found a significantly lower expression of it under hypoxia with or without TGF-*β*1 stimulation (Figure 7(a)). The 24 h stimulation led further to significantly less induction of OPN-1 under normoxic conditions. Therefore, fibroblasts seem to downregulate OPN-1* in vitro *and this may represent a negative feedback for the expression of OPN-1 during the later phases of myocardial remodeling, when extracellular matrix production is slowing down. The mRNA-expression of galectin-3 showed no differences between the strains or conditions, thus indicating no role for it in fibroblasts (Figure 7(b)). WT-myofibroblasts presented dynamic regulation of CCL2 with a significant ~4-fold higher mRNA-induction after 6 h of TGF-*β*1 stimulation irrespectively of hypoxic condition (Figure 7(c)). This induction was not observed after 24 h, while OPN^−^/^−^-hearts presented no induction of CCL2 under any of conditions. The mRNA-expression of tenascin-C was significantly induced after 6 and 24 h TGF-*β*1 stimulation and normoxia in WT-fibroblasts (Figure 7(d)). In addition, hypoxia led to a significant induction of tenascin-C mRNA after 24 h stimulation in the WT-cells. OPN^−^/^−^-hearts presented again with an absence of significant mRNA-induction of tenascin-C under any of the investigated conditions. Similar to the* in vivo* findings we observed a tendency to a higher expression of MMP-9 after 24 h TGF-*β*1 stimulation in WT-mice under hypoxia when compared to OPN^−^/^−^-cells, while this difference reached a significant level in stimulated group under normoxic conditions (Figure 7(e)). In contrast to the* in vivo *data, we found no significant induction of MMP-12 mRNA* in vitro *(Figure 7(f)). Still, another parallel to the* in vivo *data was observed in mRNA-expression of TIMP-1. TIMP-1 expression was significantly higher after 24 h TGF-*β*1 stimulation in myofibroblasts from WT-hearts under normoxic as well as hypoxic atmosphere when compared to no induction in OPN^−^/^−^-cells (Figure 7(g)).

Taken together, these data provide novel evidence for an osteopontin-mediated, differential regulation of mediators in myocardial remodeling in a model of murine ischemic cardiomyopathy.

## 4. Discussion

This study provides novel insights into cardioprotective mechanisms of osteopontin in our murine model of brief, repetitive I/R in absence of myocardial infarction. It contributes to a better understanding of the previously described role for osteopontin in regulation of macrophage function and myocardial fibrosis after infarction [6]. Osteopontin has also been associated with development of fibrosis and myocardial adaptation in an angiotensin II-induced murine hypertrophy model [9]. Mice with a cardiomyocyte specific overexpression of osteopontin died prematurely after mean of 12 weeks of age. In these mice, loss of cardiomyocytes was associated with increased inflammatory cell infiltration and their cytotoxic activity [11]. On the other hand, lack of osteopontin caused increased apoptosis of cardiomyocytes in* streptozotocin*-induced diabetic mice [16]. Further investigations showed expression of osteopontin on noncardiomyocytes in a desmin-deficient mouse model suffering from myocardial degeneration in absence of any kind of injury [12]. Expression of osteopontin has been shown on neonatal rat cardiomyocytes [17]. In the present study, we found that osteopontin is specifically induced in murine cardiomyocytes of WT-mice after repetitive ischemic insults. Our OPN^−^/^−^-mice presented with loss of cardiomyocytes and deteriorated left ventricular function after 7 days repetitive I/R. This was associated with changes in mRNA-expression of contractile elements. OPN^−^/^−^-mice did not change their utilization of the less energy efficient *α*-MHC isoform despite repetitive ischemic injury, but were still able to adapt by induction of the less ATP consuming *β*-MHC isoform. Even though this resulted in a comparable ratio of *β*/*α*-MHC between the WT and OPN^−^/^−^-mice, the increase in metabolic turnover in OPN^−^/^−^-mice may represent an additional burden under ischemic stress. The reexpression of embryonic contractile elements has been associated with different myocardial pathologies [18, 19]. Our findings on expression of skeletal and cardiac actin, as well as desmin in OPN^−^/^−^-mice, give further support to an osteopontin-mediated adaptation of cardiomyocytes to ischemic injury.

Osteopontin has been described to regulate expression of heme oxygenase-1 in glioma cells [20]. We found lower induction of heme oxygenase-1 and absent induction of glutathione peroxidase-1, as well as only minimal induction of metallothionein-1 and -2, in OPN^−^/^−^-mice, which all reflect functional impairment of antioxidative mediators as very important cardioprotective mechanism [4]. The lack of metallothionein induction in OPN^−^/^−^-mice is very interesting, since these zinc-storage proteins are important for normal function of several enzymes, antioxidative mediators, and actors in inflammatory reaction [21]. Taken together, our findings on contractile elements expression and antioxidative mediators support the specific role of osteopontin on cardiomyocytes.

According to previously published data on the role of osteopontin in macrophage function [6, 22], we found significantly stronger macrophage infiltration of the ischemic myocardium in OPN^−^/^−^-mice. In contrast to previous studies, we demonstrated predominant attraction of invading macrophages to the small, nontransmural infarctions thereby contributing to the phagocytosis of dead cardiomyocytes and subsequent scar formation. Another interesting finding of our study is the lower mRNA-induction of chemokines CCL2 and CCL3 in OPN^−^/^−^-mice during repetitive I/R. Our* in vivo* data is also supported by the lack of CCL2-induction found in our myofibroblast cell culture. These findings are novel and additive in respect to a study, where osteopontin has been suggested to specifically modulate expression of CCL2 chemokine and thus regulating activity of monocytes* in vitro* [23]. Therefore, our findings may be also interpreted as a result of osteopontin-deficiency on noncardiomyocytes, that is, macrophages and fibroblasts in the heart. But one may still argue that the lack of antioxidative mediators in cardiomyocytes could be responsible for the observed lower induction of chemokines [24]. Therefore, we can only remain speculative in this matter, and this question could be addressed using cell-specific knockouts in future studies. Despite the differences in chemokine expression, OPN^−^/^−^-mice showed normal cytokine expression during repetitive I/R, which was sufficient to mediate the observed macrophage infiltration. The resolution of inflammatory response, demonstrated by normal IL-10 mRNA-induction, also seems to be unaffected by osteopontin-deficiency, thus allowing the timely onset of myocardial remodeling. A recent study provided evidence for a substantial role of osteopontin on noncardiomyocytes, that is, macrophages via upregulation of galectin-3 and MMP-12 during myocardial degeneration of desmin-deficient mice without injury [12]. We did not find a difference in galectin-3 expression in OPN^−^/^−^-hearts, but this is probably attributable to the fact that our model of repetitive ischemic injury does not induce a substantial damage to the myocardium.

Numerous studies reported involvement of osteopontin in mechanisms of tissue remodeling. Osteopontin-expression was necessary to induce mediators of fibrosis in development of* bleomycin*-induced lung fibrosis [7]. Mice lacking osteopontin showed exaggerated left ventricular dilation and presented with reduced collagen deposition after myocardial infarction without reperfusion [10]. In contrast, we observed somewhat opposite findings with comparable total collagen area between WT- and OPN^−^/^−^-mice after 7 days I/R, but high collagen deposition in small infarctions associated with less interstitial fibrosis in the OPN^−^/^−^-mice. The collagen fibers seem not to be compacted after 7 days I/R in OPN^−^/^−^-mice, which can indicate an impaired myocardial remodeling during formation of a stable scar with compacted collagen fibers. Since osteopontin was described to be necessary for differentiation of myofibroblasts* in vitro* [8], we examined the differentiation of myofibroblasts* in vivo* and found less of these collagen-producing cells in OPN^−^/^−^-mice. Tenascin-C is an established early marker of remodeling and is also strongly involved during embryonic development [25] or scar formation after myocardial infarction [26]. In the present study, we describe for the first time that tenascin-C expression is regulated by osteopontin in myocardial remodeling* in vivo* and* in vitro*, thereby contributing to timely onset of the remodeling process. In addition to the morphological findings on less compacted collagen in small infarctions, these data clearly indicate impaired remodeling in OPN^−^/^−^-hearts. Osteopontin has also been described as an important regulator of MMPs and their tissue inhibitors during remodeling resulting in significantly decreased left ventricular dilation after myocardial infarction [27]. We could confirm these data showing a decrease in mRNA-expression of MMP-2, MMP-9, MMP-12, TIMP-1, TIMP-2, and TIMP-4* in vivo*. In addition, we found an increase in MMP-13 expression in OPN^−^/^−^-mice, being probably one of a few factors mediating the collagen deposition and scar formation during repetitive I/R in these mice. Our data from myofibroblasts* in vitro *showed a decrease in osteopontin expression under hypoxia and TGF-*β* stimulation. Therefore, osteopontin seems to be rather involved during transition from macrophage-driven inflammation to early remodeling phase, since it has also been suggested as a marker for mature macrophages during late stages of granulation tissue formation [28]. The missing upregulation of galectin-3 and MMP-12* in vitro* also points to their predominant role on macrophages, as previously suggested [12]. Still, the* in vitro *MMP-9 and TIMP-1 data are supportive for our* in vivo *findings. Therefore, our data provide also evidence for the role of osteopontin in regulation of chemokines and macrophage function and subsequent effects in remodeling during development of murine ischemic cardiomyopathy.

Our experimental study may also have a clinical relevance based on recently published human data. Osteopontin-expression was increased on cardiomyocytes in patients with terminal heart failure [29], and its level has been suggested as a predictive marker of development of right ventricular failure [30]. Also, osteopontin has been suggested as a marker of mortality and readmission risk in patients with acute congestive heart failure [31]. In this respect, we emphasize that the interpretation of our data may have a weakness being solely based on mRNA-expression without protein data. Still, this is somewhat relative due to the fact that many of our reported factors are transcriptionally well regulated.

## 5. Conclusion

In conclusion, osteopontin seems to modulate expression of contractile elements, antioxidative enzymes, inflammatory response, and early development of interstitial fibrosis in order to prevent irreversible cardiomyocyte loss in murine ischemic cardiomyopathy. These findings may further support the therapeutical perspective for osteopontin as a possible target in protection of the ischemic heart.
